# In this month’s Bulletin

**DOI:** 10.2471/BLT.19.001219

**Published:** 2019-12-01

In the editorial section, Cornelia Anne Barth (790) outlines steps needed to provide better care for 20 million people with physical disabilities living in crisis settings. Onyema Ajuebor et al. (791) introduce a curricula guide for the training of health workers in antimicrobial resistance prevention measures. Ritu Sadana et al. (792) outline the evidence needed to track the health of ageing populations.

In the news section, Andrey Shukshin and Gary Humphreys (795–796) report on drug resistant tuberculosis control programmes in Belarus. Gary Humphreys interviews Ahd Hamidi (797–798) about her work in technology transfer for vaccines. 

## Afghanistan, Brazil, Colombia, Ethiopia, Indonesia, Philippines, Peru, Thailand and Viet Nam

### Hidden costs of malaria

Angela Devine et al. (828–836) use primary trial data to extract costs per episode.

## India

### Hypertension definitions

Manisha Dubey et al. (799–809) analyse the classification effects of treatment guidelines. 

## Kenya

### Growing up with HIV

Irene Njuguna et al. (837–845) propose ways to improve the transition from pediatric to adult HIV care.

## United Kingdom of Great Britain and Northern Ireland

### Measuring impact of an industry levy

Kawther M Hashem et al. (818–827) survey caloric contents of carbonated sugar-sweetened beverages. 

## Zambia

### Surviving severe malaria

Cathy Green et al. (810–817) mobilize the community to improve outcomes. 

## Global

### Trade and nutrition talks

Pepita Barlow et al. (846–848) study the interactions between the World Health Organization and World Trade Organization.

### Paying for sustainable human development

Joseph Wong & Kimberly Skead (849–850) examine cost estimates of universal coverage.

### Access to vaccines in emergencies

Kate Elder et al. (851–853) describe a humanitarian mechanism for pneumococcal vaccines.

### Extending priorities

Lawson Ung et al. (854–856) argue that infectious corneal ulceration should be considered a neglected tropical disease.

